# Butyryl/Caproyl-CoA:Acetate CoA-transferase: cloning, expression and characterization of the key enzyme involved in medium-chain fatty acid biosynthesis

**DOI:** 10.1042/BSR20211135

**Published:** 2021-08-12

**Authors:** Qingzhuoma Yang, Shengtao Guo, Qi Lu, Yong Tao, Decong Zheng, Qinmao Zhou, Jun Liu

**Affiliations:** 1 Key Laboratory of Environmental and Applied Microbiology, Environmental Microbiology Key Laboratory of Sichuan Province, Chengdu Institute of Biology, Chinese Academy of Science, Chengdu 610041, China; 2 College of Life Sciences, University of Chinese Academy of Sciences, Beijing 100049, China; 3 Key Laboratory of Bio-Resource and Eco-Environment of Ministry of Education, College of Life Sciences, Sichuan University, Chengdu, Sichuan 610065, China; 4 BGI Education Center, University of Chinese Academy of Sciences, Shenzhen 518083, China; 5 Faculty of Bioengineering, Sichuan University of Science and Engineering, Zigong 643000, China

**Keywords:** Caproic acid, Chain elongation, CoA-transferase, Medium-chain fatty acids, Ruminococcaceae bacterium

## Abstract

Coenzyme A transferases (CoATs) are important enzymes involved in carbon chain elongation, contributing to medium-chain fatty acid (MCFA) biosynthesis. For example, butyryl-CoA:acetate CoA transferase (BCoAT) is responsible for the final step of butyrate synthesis from butyryl-CoA. However, little is known about caproyl-CoA:acetate CoA-transferase (CCoAT), which is responsible for the final step of caproate synthesis from caproyl-CoA. In the present study, two CoAT genes from *Ruminococcaceae* bacterium CPB6 and *Clostridium tyrobutyricum* BEY8 were identified by gene cloning and expression analysis. Enzyme assays and kinetic studies were carried out using butyryl-CoA or caproyl-CoA as the substrate. CPB6-CoAT can catalyze the conversion of both butyryl-CoA into butyrate and caproyl-CoA into caproate, but its catalytic efficiency with caproyl-CoA as the substrate was 3.8-times higher than that with butyryl-CoA. In contrast, BEY8-CoAT had only BCoAT activity, not CCoAT activity. This demonstrated the existence of a specific CCoAT involved in chain elongation via the reverse β-oxidation pathway. Comparative bioinformatics analysis showed the presence of a highly conserved motif (GGQXDFXXGAXX) in CoATs, which is predicted to be the active center. Single point mutations in the conserved motif of CPB6-CoAT (Asp^346^ and Ala^351^) led to marked decreases in the activity for butyryl-CoA and caproyl-CoA, indicating that the conserved motif is the active center of CPB6-CoAT and that Asp^346^ and Ala^351^ have a significant impact on the enzymatic activity. This work provides insight into the function of CCoAT in caproic acid biosynthesis and improves understanding of the chain elongation pathway for MCFA production.

## Introduction

Medium-chain fatty acids (MCFAs, C_6_–C_12_) are widely utilized in agriculture and industry. For example, *n*-caproic acid (C_6_) is used as a precursor for the production of fragrances [20], antimicrobial agents [11], and drop-in biofuels [19]. Recent studies have shown that MCFAs produced from renewable feedstock by anaerobic fermentation hold promise for replacing fossil resources and botanical oils, such as palm kernel oil, to meet the requirements for sustainable development [26]. A few microorganisms, such as *Megasphaera elsdenii* [32], *Ruminococcaceae* bacterium CPB6 [50], *Acinetobacter* spp. [21], and *Clostridium kluyveri* [46], have been reported to be able to synthesize MCFAs from renewable feedstock via the carbon chain elongation pathway [1]. In the process of chain elongation, intermediates of acidogenesis, such as acetate (C_2_) and *n*-butyrate (C_4_), act as substrates and are elongated to caproic acid (C_6_) and octanoic acid (C_8_) by addition of acetyl-CoA in reverse β-oxidation cycles [40,34]. C_2_ or C_4_, transformed to acetyl-CoA or butyryl-CoA, respectively, represents the initial substrate for elongation via reverse β-oxidation. This pathway has been identified as a key metabolic process in MCFA biosynthesis [36].

The production of high concentrations of butyrate (>10 mM) *in vitro* has been reported in some anaerobes, such as *Roseburia* [13] and *Faecalibacterium* [27]. Butyrate is normally generated from two molecules of acetyl-CoA, yielding acetoacetyl-CoA, which is then converted into butyryl-CoA [47]. In the latter reaction, butyryl-CoA is exchanged with exogenously derived acetate to yield acetyl-CoA and butyrate [35]. The enzymes responsible for butyrate production in the reverse β-oxidation pathway are acetyl-CoA acetyltransferase (AtoB, EC 2.3.1.9), 3-hydroxybutyryl-CoA dehydrogenase (Hbd, EC 1.1.1.157), enoyl-CoA hydratase (Crt, EC 4.2.1.17), butyryl-CoA dehydrogenase (Bcd, EC 1.3.2.1), and butyryl-CoA:acetate CoA transferase (BCoAT, EC 2.8.3.8) [18]. Among them, BCoAT is a well-known coenzyme A transferase (CoAT) responsible for the final step of butyric acid synthesis, transforming the CoA moiety from butyryl-CoA to an exogenous acetate molecule, which results in the formation of butyrate and acetyl-CoA [17,8]. CoATs are abundant in anaerobic fermenting bacteria that cope with low ATP yields, but they are also found in aerobic bacteria and in the mitochondria of humans and other mammals [44]. The synthesis pathway and key genes associated with butyric acid in MCFA biosynthesis via reverse β-oxidation are well understood. However, little is known about key genes involved in the conversion of butyric acid (C_4_) into caproic acid (C_6_). Although most genes responsible for butyric acid production are suggested to function in further chain elongation of MCFAs [18], the fact that many butyrate-producing bacteria, such as *Clostridium tyrobutyricum*, produce only butyric acid instead of caproic acid via the reverse β-oxidation pathway suggests that there may be different functional genes involved in the production of caproic acid.

Recently, our study showed that *Ruminococcaceae* bacterium CPB6 is a caproic acid-producing bacterium with a highly prolific ability to perform chain elongation and can produce caproic acid (C_6_) from lactate (as an electron donor) with C_2_–C_4_ carboxylic acids and heptanoic acid (C_7_) with C_3_–C_5_ carboxylic acids as electron acceptors (EAs) [51,45]. Moreover, a set of genes correlated with chain elongation were identified by sequencing and annotating the entire genome of the CPB6 strain [48]. However, very little information is available on enzymes involved in the conversion of C_4_ into C_6_, especially the gene responsible for the conversion of caproyl-CoA into caproic acid.

In the present study, we cloned a predicted caproyl-CoA:acetate CoA-transferase (CCoAT) gene from the caproic acid-producing strain CPB6 [51] and a BCoAT gene from the butyric acid-producing *C. tyrobutyricum* BEY8 and expressed the two proteins in *Escherichia coli* BL21 (DE3) using pET28a. The aims of the present study were to: (i) compare differences in sequence, structure, enzymatic activity, and substrate specificity between CCoAT and BCoAT; (ii) identify the active center of CCoAT and its effects on the activities of enzymes with different structures; and (iii) verify the existence of CCoAT in the caproic acid biosynthesis pathway.

## Materials and methods

### Strain growth conditions

*E. coli* DH5α (TsingKe, Chengdu, China) and *E. coli* BL21 (DE3) (Transgene, Beijing, China) were cultured in Luria broth (LB) medium supplemented with 50 µg/ml kanamycin (Sangon Biotech, Shanghai, China) at 37°C. *Ruminococcaceae* bacterium CPB6 was grown anaerobically at 37°C in modified reinforced *Clostridium* medium (Binder, Qingdao, China). *C. tyrobutyricum* BEY8 was grown anaerobically at 37°C in TGY medium (30 g/l tryptone, 20 g/l glucose, 10 g/l yeast extract, and 1 g/l l-cysteine hydrochloric acid; pH 7).

### Gene cloning and plasmid construction

The CoAT genes were amplified from the genomic DNA of *Ruminococcaceae* bacterium CPB6 [48] or *C. tyrobutyricum* BEY8 [22] through PCR using the primers listed in Table 1. During amplification, the following conditions were used: initial denaturation (5 min at 98°C); followed by 30 cycles of denaturation (10 s at 98°C), annealing (30 s at 52°C), and elongation (1 min at 72°C); and a final extension (5 min at 72°C). The PCR products were verified by agarose gel electrophoresis, recovered using a PCR purification kit (Fuji, Chengdu, China), and seamlessly inserted into pET28a double digested with *Not* I and *Sal* I (Thermo, Waltham, U.S.A.) to construct the recombinant plasmid using a seamless cloning kit (Biomed, Beijing, China). The recombinant plasmids were verified by Sanger sequencing and then used to transform *E. coli* BL21 (DE3) cells.

**Table 1:** Bacterial strains, plasmids, and primers used in the present study

	Description	Reference or source
Strains		
CPB6	*Ruminococcaceae* bacterium CPB6	Zhu et al.
BEY8	*C. tyrobutyricum* BEY8	Hu et al.
*E. coli* DH5α	TreliefTtMm 5α chemically competent cells	TsingKe
*E. coli* BL21 (DE3)	Expression chemically competent cells	Transgene
Plasmids		
pET28a	*E. coli* expression vector (Kan, T7 promoter)	The present study
pET28a-CoAT-CPB6	pET28a carrying the CoAT gene from CPB6 fused with a His tag at the N-terminus	The present study
pET28a-CoAT-BEY8	pET28a carrying the CoAT gene from BEY8 fused with a His tag at the N-terminus	The present study
D346H-mutant	pET28a-CoAT-CPB6 with the Asp^346^ aa mutation in CoAT	The present study
A351P-mutant	pET28a-CoAT-CPB6 with the Ala^351^ aa mutation in CoAT	The present study
**Primers**	**Sequence (5′–3′)**	
YT43-SalΙ-fw	TAATACGACTCACTATAGGG	The present study
YT44-NotΙ-rv	GCTAGTTATTGCTCAGCGG	The present study
YT50-CPB6-fw^1^	GTGGTGCTCGAGTGCATGAGTTTTCAAGAAGAATATGCACAAAAACTGAC	The present study
YT51-CPB6-rv^1^	CGAATTCGAGCTCCGTTAAATTTTATTGCTTCTGCGCCAGATGC	The present study
YT52-BEY8-fw^1^	CGAATTCGAGCTCCGTCGACATGAGTTTTGAGGAATTGTATAAGAGTAAAGTTGTTAGT	The present study
YT53-BEY8-rv^1^	GTGGTGCTCGAGTGCGGCCGCTTTTATAAGTTCTCTAGCTCTTTGTTTTAATGTCTTACCTCTAAG	The present study
YT60-A351P-fw^2^	AGCTGGATTTTGTTCTGGGTCCCTATCTGAGCCACGGT	The present study
YT61-A351P-rv^2^	GACCCAGAACAAAATCCAGCTGACCGGCAC	The present study
YT62-D346H-fw^2^	AGCGGTGCCGGTGGTCAGCTGCATTTTGTTCT	The present study
YT63-D346H-rv^2^	GCAGCTGACCACCGGCACCGCTAATCTGACGAAA	The present study

1 Underscored letters match the sequence of vectors for seamless cloning.

2 The sequences corresponding to the mutated codons are written in bold.

### Expression and purification of the CoA-transferases

*E. coli* BL21 (DE3) were transformed with the recombinant plasmids pET28-CoAT-CPB6 (pET28-CCoAT) and pET28-CoAT-BEY8 (pET28-BCoAT). The transformed cells were cultured in LB medium containing 50 µg/ml kanamycin at 37 °C until the OD_600_ reached 0.5 and then further cultured at 22 °C for 12 h with 0.4 mM IPTG. The cultured cells were harvested by centrifugation (8000×***g***, 10 min) at 4°C, and the cell pellet was resuspended in 50 mM potassium phosphate (pH 8.0). The cells were then disrupted with an ultrasonicator (Huxi, Shanghai, China) for 30 min (200 W, 4 s, interval 6 s) and centrifuged at 8000×***g*** for 30 min to remove the insoluble material. Then, the enzyme was purified with Ni-NTA Sepharose (Genscript, Nanjing, China) and eluted with 50 mM sodium phosphate (pH 8) containing 300 mM NaCl and 250 mM imidazole. Finally, the purity and MW of the enzyme were assessed using SDS/PAGE analysis. Moreover, the enzyme was analyzed via Western blotting with anti-6× His rabbit polyclonal antibody (Sangon Biotech, Shanghai, China) to determine whether the target protein was obtained. The protein concentrations were determined using a BCA protein assay kit (Solarbio, Beijing, China).

### Enzymatic characterization

The CoAT activity in crude enzyme extracts and of purified recombinant proteins was measured by determining the concentration of acetyl-CoA, a reaction byproduct, using a citrate synthase assay described in previous studies with minor modifications [37,25]. In brief, the reaction was initiated by the addition of enzyme (up to 20 ng/ml) and was performed in a total volume of 1 ml at 25°C: 100 mM potassium phosphate buffer (pH 7), 200 mM sodium acetate, 1 mM 5,5′-dithiobis(2-nitrobenzoate), 1 mM oxaloacetate, 8.4 *n*kat citrate synthase (Sigma, St. Louis, U.S.A.), and 0.5 mM CoA derivatives (Sigma, St. Louis, U.S.A.). The released CoA, corresponding to the formed amount of acetyl-CoA, was detected by measuring the absorbance at 412 nm. One unit of activity is defined as the amount of enzyme that converts 1 µmol of acetyl-CoA per min under these conditions.

The kinetic parameters of the recombinant protein were also calculated by using a coupled spectrophotometric enzyme assay through citrate synthesis [35]. The reaction mixture was the same as that mentioned above, and the concentrations of butyryl-CoA or caproyl-CoA were varied from 0.5 to 5 mM. The kinetic parameters were computed using the Lineweaver–Burk transformation of the Michaelis–Menten equation, in which velocity is a function of the substrate [5,6]. The catalytic constant (*k*_cat_) was defined as the number of CoAT molecules formed by one molecule of enzyme in a single second. All measurements were performed in triplicate for each biological replication.

### Sequence alignment and phylogenetic reconstruction

Multiple alignment of CoAT amino acid sequences was performed using ESPript [9]. With the Akaike information criterion (AIC), the amino acid substitutions were predicted using ProtTest (version 3.4.2). We constructed a phylogenetic tree of the whole genomes of strains containing CoAT [20,12,3]. The construction followed the general approach of [15,49] and employed sequences downloaded from the online database NCBI (https://www.ncbi.nlm.nih.gov/). MEGA-X software was used to construct the whole genome phylogenetic tree [43]. The phylogenetic relationships of CoATs from different species were obtained by using OrthoFinder [15], and MUSCLE (version 3.8.31) was used to calibrate the 119 shared single-copy genes [14]. The phylogenomic tree was derived from a supermatrix comprising these shared single-copy genes with 41213 unambiguously aligned amino acids using the maximum likelihood (ML) method in RAxML (version 8.2.10) [41] under the PROTGAMMAAUTO model, with 100 bootstrap replicates.

### Prediction of tertiary structures of CoAT proteins

The online modeling tool ScanProsite was used for protein homology comparisons, and SWISS-MODEL [2] was used to predict the active sites, tertiary structures and corresponding functions of CoAT proteins in multiple strains (*Ruminococcaceae* bacterium CPB6, *Clostridium kluyveri, Megasphaera elsdenii, Clostridium tyrobutyricum* BEY8, *Lachnospiraceae* bacterium, and *Anaerostipes hadrus*) to confirm possible variations in the tertiary structures. The targeted sequence was uploaded to search for the best matched template on the basis of data coverage and identity. We subsequently conducted model–template alignment for structural comparisons. Finally, the predicted CoAT protein tertiary structures were embellished and labeled using PyMOL (version 2.3.3) [31] and were adjusted in a similar pattern to identify variations.

### Identification of putative positively selected sites

According to the above analysis, relatively conserved regions were identified, and the differences in properties and structures among different amino acids were compared with find two amino acids that might be key sites for site-directed mutagenesis. The point mutation vectors were constructed with the Fast Mutagenesis System (Transgene, Beijing, China). The QuikChange PCR method using pfu DNA polymerase was performed to generate the D346H mutant and A351P mutant. The recombinant plasmid (pET28a-CoAT-CPB6) was used as template DNA, and the complementary mutagenic oligonucleotides used as primers are shown in Table 1. After PCR amplification, the mixture was digested with restriction enzymes using D*pn*I to remove methylated template DNA and then sequenced (TsingKe, Chengdu, China) to verify site mutagenesis before being used to transform *E. coli* BL21 (DE3) (Transgene, Beijing, China). After purification, the enzymatic activities in the presence of butyryl-CoA and caproyl-CoA were measured following the method described above for the wildtype.

## Results

### Cloning, expression, and purification of CoA-transferase

According to the genome sequences of strains CPB6 and *C. tyrobutyricum* BEY8, specific primers targeting CoAT genes were designed and synthesized (Table 1). Agarose gel electrophoresis showed that the size of the PCR products and the double-digestion products was approximately 1300 bp, consistent with the expected sizes of the CPB6-CoAT (1344 bp) (Supplementary Figure S1) and the BEY8-CoAT (1233 bp) genes (Supplementary Figure S2). Sequence analysis of the recombinant CoAT plasmids showed that the cloned genes shared 100% similarity with the predicted CoAT genes of strains CPB6 (a CCoAT) and BEY8 (a BCoAT). This finding indicated that the recombinant *E. coli*/pET28a-CCoAT and *E. coli*/pET28a-BCoAT were successfully constructed.

**Figure 1:**
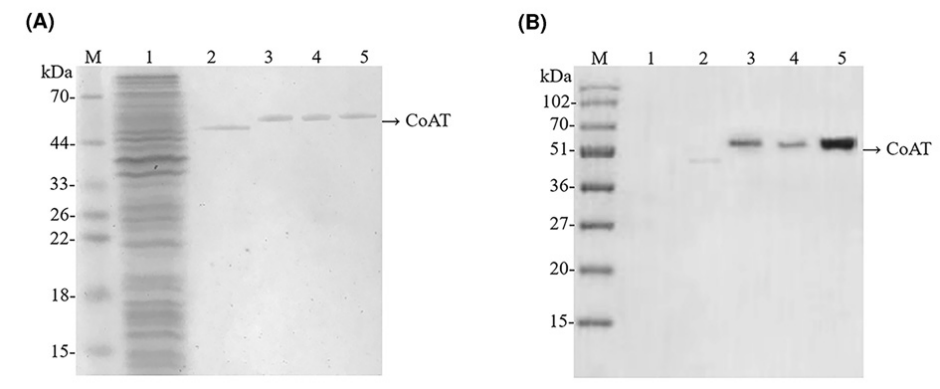
Purification and Western blot analysis of CPB6-CoAT (a CCoAT) and BEY8-CoAT (a BCoAT) Analysis of purified CCoAT and BCoAT via SDS/PAGE (**A**). Analysis of purified CCoAT and BCoAT via Western blotting with an anti-His-tag antibody (**B**). M, molecular mass marker. Lanes: 1, pET28a; 2, BCoAT; 3, CCoAT; 4, CCoAT-D346H mutant; 5, CCoAT-A351P mutant. Samples (∼2 µg) were visualized by Coomassie Brilliant Blue staining after electrophoresis. Molecular mass positions are shown by markers (kDa).

To characterize the functions of CoAT proteins, two recombinant plasmids (pET28a-CCoAT and pET28a-BCoAT) were expressed in *E. coli* BL21 (DE3). Single bands of the purified proteins were detected on SDS/polyacrylamide gels after affinity chromatography (Figure 1A). As shown in Figure 1A, there was no obvious protein band approximately the size of the target protein in *E. coli*/pET28a (control), while a single band was observed in *E. coli*/pET28a-BCoAT (lane 2) and *E. coli*/pET28a-CCoAT (lane 3), and the band sizes were consistent with the expected sizes of BEY8-CoAT (46 kDa) and CPB6-CoAT (49 kDa). Furthermore, Western blotting analysis with a His antibody (Figure 1B) also demonstrated that the observed bands were consistent with the expected molecular mass of BEY8-CoAT and CPB6-CoAT (approximately 46–49 kDa).

### Enzyme assay

The CoAT activity of crude enzyme extracts was determined by measuring the production of acetyl-CoA from butyryl-CoA or caproyl-CoA [37]. Previously, the key reactions for butyrate and caproate production have been reported to be (1) butyryl-CoA + acetate → butyrate + acetyl-CoA and (2) caproyl-CoA + acetate → caproate + acetyl-CoA [40,16,51]. As shown in Table 2, the crude and purified BEY8-CoAT activities with butyryl-CoA and sodium acetate as substrates were 6.91 ± 0.12 and 26.2 ± 0.09 U/mg of protein, respectively. However, this enzyme showed no activity for caproyl-CoA. This result suggests that BEY8-CoAT is a BCoAT, similar to the CoAT from *Clostridium acetobutylicum* ATCC 824 that is able to produce butyrate instead of caproate, and its purified enzyme activity was 29.1 U/mg of protein [10]. Moreover, the butyrate-producing bacterium *Coprococcus* sp. strain L2-50 from the human large intestine showed very high BCoAT activity (118.39 ± 5.02 U/mg of protein) but no CCoAT activity [13]. This result indicates that the BCoAT probably has substrate specificity for butyryl-CoA. In contrast, the activities of crude and purified CPB6-CoAT with butyryl-CoA and sodium acetate as substrates were 2.07 ± 0.06 and 10.8 ± 0.02 U/mg of protein, and the activities with caproyl-CoA and sodium acetate as substrates were 5.11 ± 0.08 and 27.6 ± 0.15 U/mg of protein, respectively (Table 2), indicating that CPB6-CoAT can catalyze the conversion of both butyryl-CoA into butyrate and caproyl-CoA into caproate. Notably, the crude and purified CPB6-CoAT activity for caproyl-CoA was 2.5–2.6-times higher (5.11 vs 2.07, 27.56 vs 10.28 U/mg of protein) than that for butyryl-CoA, suggesting that CPB6-CoAT specifically prefers caproyl-CoA as a substrate instead of butyryl-CoA.

**Table 2:** Purification and specific activities of the CoATs^1^

Enzyme	Total protein, mg	Total activity, U	Specific activity, U/mg of protein		Purification fold
			Butyryl-CoA	Caproyl-CoA	
CPB6-CoAT					
Crude extract	87.04	180.2	2.07 ± 0.06	5.11 ± 0.08	1
Purified protein^2^	22.61	244.2	10.8 ± 0.02	27.6 ± 0.15	5.4
BEY8-CoAT					
Crude extract	63.10	436.0	6.91 ± 0.12	ND^1^	1
Purified protein^2^	17.66	462.7	26.2 ± 0.09	ND^1^	3.8

1 The purification data in the table were obtained from 300 ml of culture medium. Abbreviations: ND, not detectable; U, μmol/min.

2 Protein was purified via affinity chromatography.

### Kinetics of CoA-transferases

The kinetic parameters of the recombinant proteins were investigated using a colorimetric assay according to a previous study [35]. Initial velocities were determined at fixed sodium acetate concentrations with different butyryl-CoA or caproyl-CoA concentrations. *K*_m_ and *V*_m_ values were estimated from secondary plots (‘Materials and methods’ section). Additionally, *k*_cat_ values were calculated from enzyme concentrations in the reaction mixtures. The double-reciprocal enzyme kinetics plot showed that the reactions of the two CoATs follow a ternary-complex mechanism (Supplementary Figure S4).

As *k*_cat_/*K*_m_ can be used to compare the catalytic efficiency of different substrates catalyzed by the same enzyme [24], a lower *K*_m_ value indicates that the enzyme has a higher affinity for the substrate, and *vice versa* [30]. In this study, the *K*_m_, *k*_cat_ and *k*_cat_/*K*_m_ values for CPB6-CoAT with caproyl-CoA were 359 ± 5.3 µM, 14.7 ± 0.9 min^−1^ and 41.1 ± 0.2 mM^−1^.min^−1^, respectively, and those with butyryl-CoA were 537 ± 10 µM, 5.81 ± 1.5 min^−1^ and 10.8 ± 0.2 mM^−1^.min^−1^, respectively (Table 3). The catalytic efficiency of CPB6-CoAT for caproyl-CoA was 3.8-times (41.1 ± 0.2 vs 10.8 ± 0.2 mM^−1^.min^−1^) higher than that for butyryl-CoA, consistent with our previous result showing that the CCoAT activity is predominantly higher than the BCoAT activity [51]. The *K*_m_ of CPB6-CoAT for caproyl-CoA was significantly lower than that for butyryl-CoA (359 ± 5.3 vs 537 ± 10 µM), illustrating the higher affinity of this enzyme for caproyl-CoA relative to butyryl-CoA. These results also partly explain why caproate instead of butyrate is always the predominant product in the fermentation broth of strain CPB6 [48,45]. BEY8-CoAT had only BCoAT activity, with *K*_m_, *k*_cat_ and *k*_cat_/*K*_m_ values of 370 ± 4.1 µM, 13.9 ± 0.7 min^−1^ and 37.7 ± 0.2 mM^−1^.min^−1^, respectively, and there was no detectable CCoAT activity (Tables 2 and 3), supporting our previous results showing that strain BEY8 produces only butyric acid as the predominant product. Statistical gap analysis of the above enzymatic experimental data showed *P*-values that were less than 0.01, indicating a significant difference between them. Similar to the results of Lee et al. [22], the CoAT from *C. tyrobutyricum* only catalyzes the conversion of butyryl-CoA into butyrate and is not responsible for chain elongation of larger or higher carbon-numbered (>C_5_) fatty acids.

**Table 3:** Kinetic parameters for the CoATs

Enzyme	Butyryl-CoA			Caproyl-CoA			Reference
	*K*_m_ (μM)	*k*_cat_ (min^−1^)	*k*_cat_/*K*_m_ (mM^−1^.min^−1^)	*K*_m_ (μM)	*k*_cat_ (min^−1^)	*k*_cat_/*K*_m_ (mM^−1^.min^−1^)	
BEY8-CoAT	370 ± 4.1	13.9 ± 0.7	37.7 ± 0.2	ND^3^	ND^3^	ND^3^	The present study
CPB6-CoAT	537 ± 10	5.81 ± 1.5	10.8 ± 0.2	359 ± 5.3	14.7 ± 0.9	41.1 ± 0.2	The present study
CPB6-CoAT-D346H-mutant	747 ± 2.8	1.30 ± 0.2	1.73 ± 0.1	748 ± 7.9	4.24 ± 0.2	5.66 ± 0.1	The present study
CPB6-CoAT-A351P-mutant	623 ± 4.4	2.99 ± 0.9	4.80 ± 0.2	532 ± 2.5	6.78 ± 1.2	12.8 ± 0.5	The present study
PGN_0725^1^	520 ± 10	9.33 ± 0.7	17.95 ± 0.1	NR^3^	NR^3^	NR^3^	[35]
CoA transferase^2^	21.0 ± 0.1	NR^3^	NR^3^	NR^3^	NR^3^	NR^3^	[10]

1 Butyryl-CoA:acetate CoA transferase from *Porphyromonas gingivalis*.

2 Butyryl-CoA:acetate CoA transferase from *Clostridium acetobutylicum* ATCC 824.

3 ND is defined as not determined. Abbreviation: NR, not reported.

### Phylogenetics of the whole genome and multiple amino acid sequence alignment

**Figure 2:**
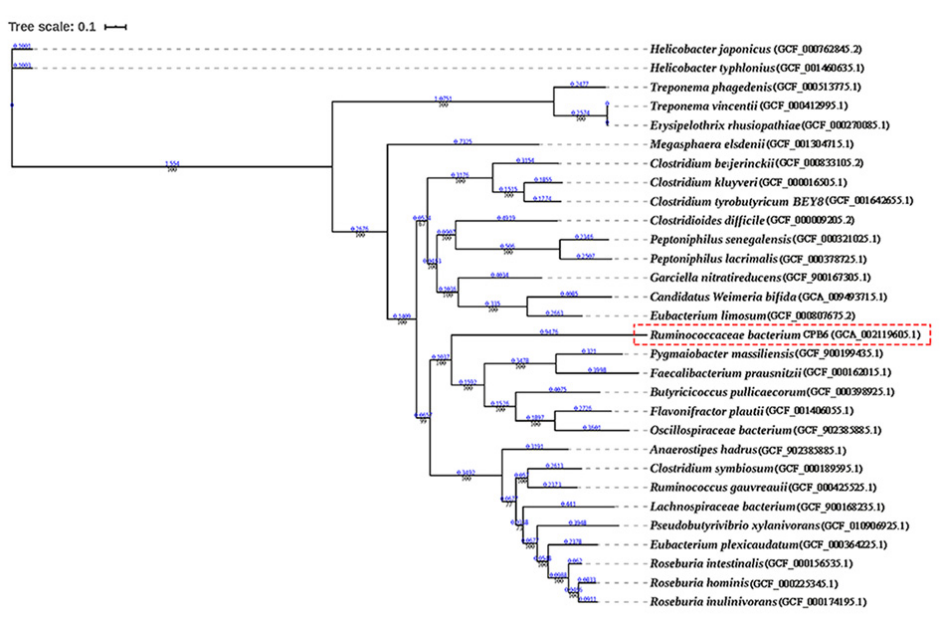
Phylogenetic tree of the whole genomes of 29 strains containing the CoA-transferase Numbers at the nodes indicate the levels of bootstrap values. The scale bar for the tree represents a distance of 0.1 substitutions per site.

A phylogenetic tree of CoATs from different strains was constructed, as shown in Supplementary Figure S5. The whole-genome phylogenetic tree was constructed based on 119 single-copy genes (including CoATs) that were common among 29 strains (Figure 2). These strains have a wide range of butyrate metabolic pathways [28], for example, *Roseburia* sp., *Faecalibacterium prausnitzii*, and *Coprococcus* sp. from the human gut exhibit BCoAT activity values of 38.95, 18.64, and 118.39 U/mg of protein (crude extracts), respectively [13]. The two species closest to strain CPB6 were *Pygmaiobacter massiliensis* [4] and *F. prausnitzii* [39], which are also butyric acid-producing bacteria in human feces. Interestingly, the species closest to *C. tyrobutyricum* BEY8 was *C. kluyveri*, which is a well-known caproic acid-producing bacterium. This close relationship may be because they belong to the same genus, *Clostridium*.

**Figure 3:**
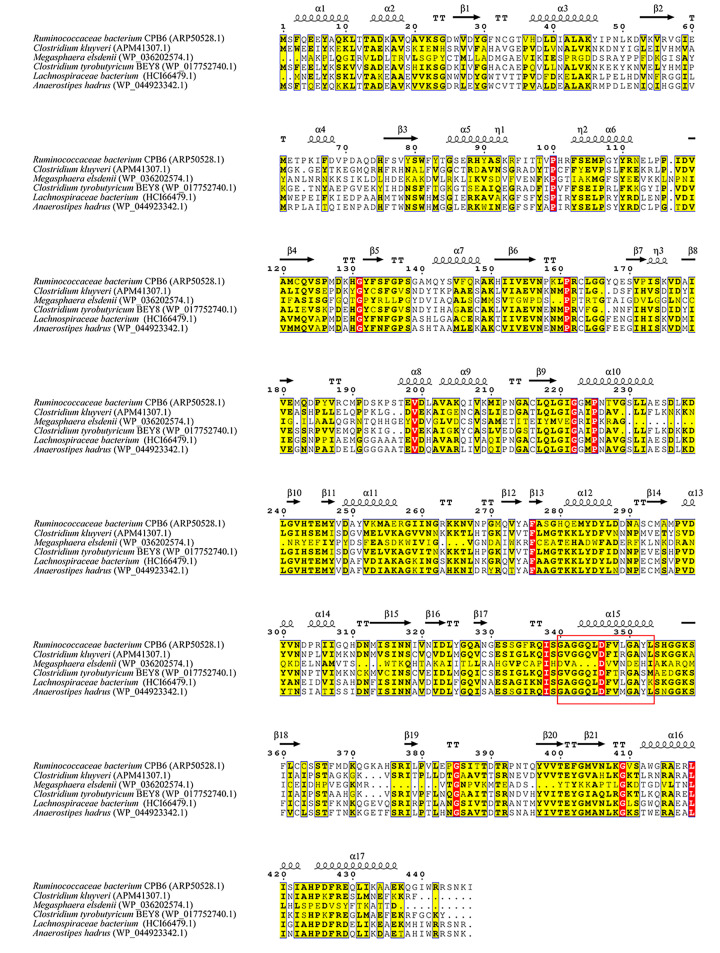
Multiple amino acid sequence alignment for CoATs The structure contained 17 α-helices and 21 β-pleated sheets, which are represented with symbols. Nonconserved, 60% conserved, and 100% conserved residues are marked with white, yellow, and red font, respectively. Conserved motifs are boxed with a red frame.

Based on the alignment results generated from CoAT protein sequences from six different species, 14 amino acids (GXGGQXDFXXGAXX, positions 340–353) of the CoATs in all the microbes were highly conserved (except in *M. elsdenii*), and their secondary structures consisted of 17 α-helices and 21 β-sheets (Figure 3). The sequence similarities between CPB6-CoAT and the analyzed CoATs were as follows: *C. kluyveri* (37.67%), *M. elsdenii* (10.27%), *C. tyrobutyricum* BEY8 (38.04%), *Lachnospiraceae* bacterium (60.59%), and *Anaerostipes hadrus* (58.52%). Among the six bacteria, strains CPB6, *C. kluyveri*, and *M. elsdenii* are caproic acid-producing bacteria, while *C. tyrobutyricum* BEY8, *Lachnospiraceae* bacterium, and *A. hadrus* are butyric acid-producing bacteria. The alignment results showed that CPB6-CoAT shared lower similarity (10.27–37.67%) with the CoATs of *C. kluyveri* and *M. elsdenii* and higher similarity (58.52–60.59%) with the CoATs of *Lachnospiraceae* bacterium and *A. hadrus*. This may be because strain CPB6 belongs to the family *Ruminococcaceae*, which is closer to *Lachnospiraceae* and *Anaerostipes* at the taxonomic phylogeny level than to *Megasphaera* and *Clostridium*.

### Prediction and comparison of the three-dimensional structure and active site

**Figure 4:**
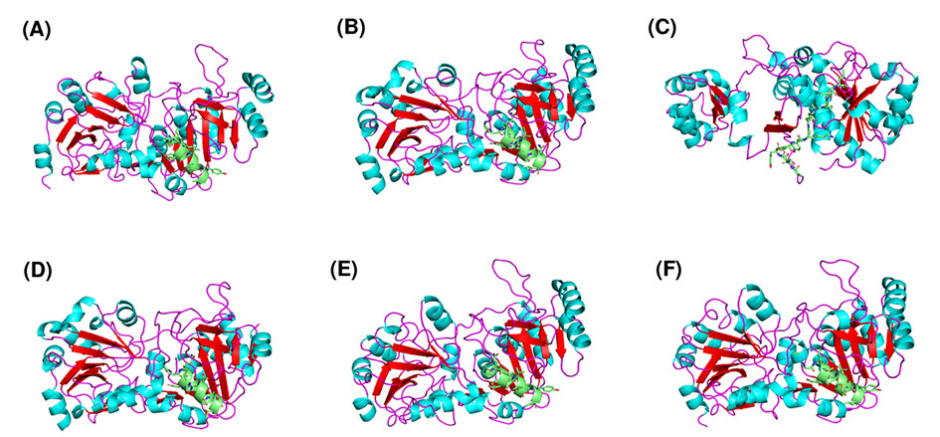
Predicted 3D structures of representative CoAT proteins 3D structures of CoATs from *Ruminococcaceae* bacterium CPB6 (**A**), *C. kluyveri* (**B**), *M. elsdenii* (**C**), *C. tyrobutyricum* BEY8 (**D**), *Lachnospiraceae* bacterium (**E**), and *A. hadrus* (**F**). Helices of the catalytic domains, β-pleated sheets, loop regions, and active centers are colored sky blue, red, purple, and green, respectively.

**Figure 5:**
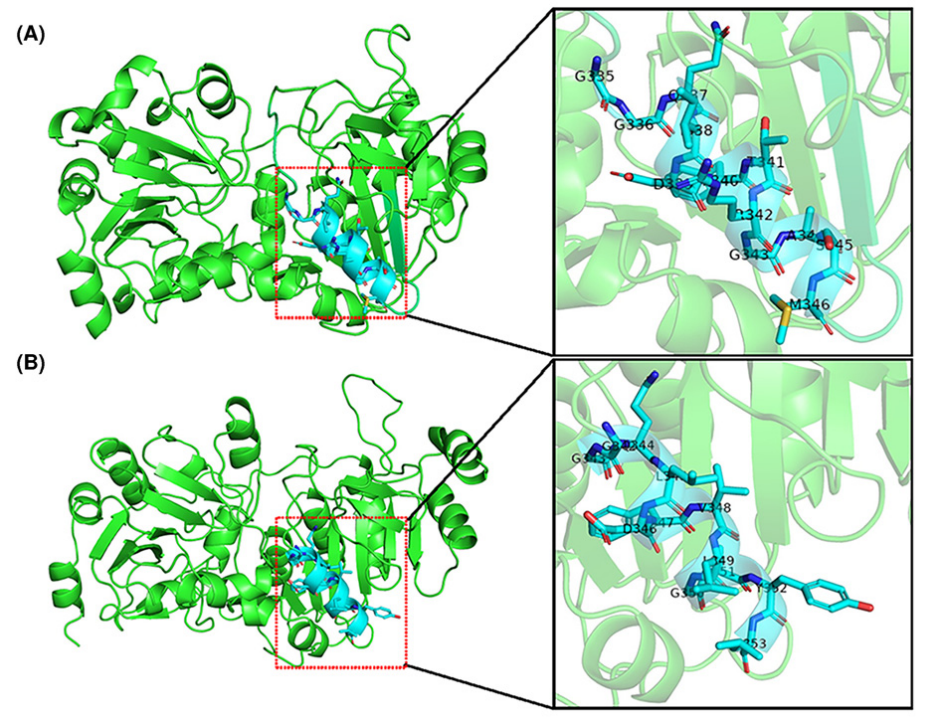
Predicted 3D structures of the CoAT proteins (left) and the active center (right) (**A**) BEY8-CoAT; (**B**) CPB6-CoAT.

As shown in Figure 4A, the three-dimensional (3D) structure of CPB6-CoAT has one subunit that may consist of two main domains, resulting in a characteristic two-domain fold in a homotetrameric structure. A comparison of the 3D structures of the six CoAT proteins (Figure 4) showed that these CoATs shared similar conformations of their structural elements (α-helices and β-strands) with slight structural modifications in the loop regions and active centers, with the exception of the CoAT from *M. elsdenii* (Figure 4C). The 3D structure of the *M. elsdenii* protein was obviously different from that of other CoATs, and the divergences were located not only in the structural elements of α-helices and β-strands but also in the loops. This may be attributed to the distant genetic relationship between *M. elsdenii* and the other five bacteria. Although *M. elsdenii* produces caproic acid via acetyl-CoA and succinate [23], the functions of the CoATs may differ between strain CPB6 and *M. elsdenii*. The 3D structures of CoATs among *C. tyrobutyricum* BEY8, *Lachnospiraceae* bacterium, and *A. hadrus* shared almost the same conformation of α-helices and β-strands except for some slight variation in the loops (Figure 4D–F). The protein structure and active center structures were further compared between CPB6-CoAT and BEY8-CoAT, as shown in Figure 5, and both showed similar 3D structures except for the location and structure of the active center. The predicted active sites of the six CoATs are shown in Table 4. The predicted active center of the CPB6-CoAT protein was located between amino acids 342 and 353 (GGQLDFVLGAYL), while the active center of the BEY8-CoAT protein (GGQIDFTRGASM) was located at amino acids 335–346, and both active sites contained phenylalanine and tyrosine (Figure 5).

**Table 4:** Prediction of the active sites of CoATs in different strains

Strain	Location of active site	Sequence of active site
*Ruminococcaceae* bacterium CPB6 (ARP50528.1)	342–353	GGQLDFVLGAYL
*C. kluyveri* (APM41307.1)	335–346	GGQVDFIRGANL
*M. elsdenii* (WP_036202574.1)	356–367	ADSYTYKKAPTL
*C. tyrobutyricum* BEY8 (WP_017752740.1)	335–346	GGQIDFTRGASM
*Lachnospiraceae* bacterium (HCI66479.1)	340–351	GGQLDFVLGAYK
*A. hadrus* (WP_044923342.1)	342–353	GGQLDFVMGAYL

### Site-directed mutagenesis

Site-directed mutagenesis was used to verify the active site of the proteins [38]. According to the predicted active center of CPB6-CoAT (GGQLDFVLGAYL, 342–353 aa) and compared with others (Table 4), site-directed mutagenesis targeting Asp^346^ and Ala^351^ was carried out to identify the effects of the two residues on the catalytic activity of CPB6-CoAT. Specifically, Asp^346^ was replaced by His and Ala^351^ was replaced by Pro via site-directed mutagenesis. The nucleotide substitutions were confirmed by Sanger sequencing of the DNA (Supplementary Figure S6). Enzyme assays showed that compared with wildtype CPB6-CoAT, the Asp^346^ substitution led to an approximately 76% loss of BCoAT activity and 72% loss of CCoAT activity, while the Ala^351^ substitution resulted in an almost 50% loss of BCoAT activity and 55% loss of CCoAT activity (Supplementary Figure S7). Statistical gap analysis of the above enzymatic experimental data (*P*-values <0.01) indicated a significant difference between them. The initial velocity of the reaction in different samples at different substrate concentrations can be seen in Supplementary Figure S8. Moreover, as shown in Table 3, the *k*_cat_/*K*_m_ values for the D346H mutant with butyryl-CoA and caproyl-CoA (1.73 ± 0.1 and 5.66 ± 0.1 mM^−1^.min^−1^) and the A351P mutant (4.80 ± 0.2 and 12.8 ± 0.5 mM^−1^.min^−1^) were significantly lower than that for the wildtype CPB6-CoAT (10.8 ± 0.2 and 41.1 ± 0.2 mM^−1^.min^−1^), indicating that the Asp^346^ and Ala^351^ residues have significant effects on the enzyme activity, but the effect of Ala on the enzyme activity was lower than that of Asp.

## Discussion

In the reverse β-oxidation pathway contributing to MCFA biosynthesis, BCoAT is required for butyrate biosynthesis in *C. kluyveri* [13] and *C. tyrobutyricum* [22]. This enzyme is responsible for the final step of butyrate production, catalyzing the conversion of butyryl-CoA and acetate into butyrate and releasing acetyl-CoA [35]. As reported in previous studies, this enzyme is considered to be a biomarker for identifying butyrate-producing bacteria [10,28,35] and may be involved in the conversion of caproyl-CoA into caproate in *C. kluyveri*, similar to the conversion of butyryl-CoA into butyrate [18]. In our present study, the BCoAT from *C. tyrobutyricum* only has activity for butyryl-CoA but has no activity for caproyl-CoA, suggesting that BCoAT in *C. tyrobutyricum* is not responsible for chain elongation of larger or higher carbon-numbered (>C_5_) fatty acids [22]. Moreover, the *K*_m_ of BEY8-CoAT for butyryl-CoA (370 ± 4.1 µM) was obviously greater than that of CPB6-CoAT (537 ± 10 µM), indicating that BEY8-CoAT had a higher enzymatic affinity for butyryl-CoA than CPB6-CoAT. Similarly, the CoAT (PGN_0725) from *Porphyromonas gingivalis* [35,47] and CoAT from *C. acetobutylicum* ATCC 824 [10] both catalyze the conversion of butyryl-CoA into butyrate, with *K*_m_ values of 520 ± 10 and 21.0 ± 0.1 µM, respectively. This indicates that BCoAT generally has higher affinity and catalytic activity for butyryl-CoA than CCoAT, while no BCoAT from butyric acid bacteria displayed affinity and catalytic activity for caproyl-CoA. These results suggest that BCoAT is only involved in chain elongation of C_2_–C_4_, not in that of C_4_ to C_6_ or C_8_.

Our previous study showed that the rate of caproate production with caproyl-CoA as the substrate in strain CPB6 was 3.5-times higher than that observed with butyryl-CoA as the substrate and suggested the existence of a CCoAT that specifically prefers caproyl-CoA instead of butyryl-CoA as the substrate [51]. In this study, CPB6-CoAT was confirmed for the first time to be a CCoAT responsible for the final step of caproate formation, although it has low BCoAT activity for butyryl-CoA. These data demonstrated the existence of a specific CCoAT involved in the chain elongation of MCFAs, which is significantly different from the function of BCoAT. The CPB6-CoAT protein catalyzed transferase reactions via a ternary-complex kinetic mechanism, whereas some other CoA transferases from *Acidaminococcus fermentans* [6], *C. acetobutylicum* ATCC 824 [10] and *Clostridium propionicum* [38], which belong to family I transferases, were reported to catalyze a transferase reaction via a ping-pong bi–bi mechanism. Thus, CPB6-CoAT was different from them in terms of substrate specificity and kinetic mechanism. The detailed mechanism underlying this functional difference needs to be further studied.

The structure of proteins plays an important role in their functional properties and catalytic efficiency [52]; for example, succinyl CoA:3-ketoate CoA transferase from pig heart [42] and 4-hydroxybutyrate CoA-transferase from *Clostridium aminobutyricum* [29] showed unexpected changes in protein modification and specific activity when their crystal structures changed. In the present study, a comparison of the 3D and active center structures showed similarities and differences between CPB6-CoAT and other CoATs (Figure 4 and Supplementary Figure S5), which may have affected enzyme catalytic function and activity. On the basis of these results, studying the functional differences caused by the structural changes in CoATs is of great significance. The exact structure and function of the active center of the CPB6-CoAT protein remains to be determined through subsequent comprehensive experiments and analysis. Additionally, site-directed mutagenesis showed that two residues (Asp^346^ and Ala^351^) in the conserved motif (GGQLDFVLGAYL, 342–353 aa) had significant effects on the enzymatic activity of CPB6-CoAT, but the effect of Ala^351^ on the enzyme activity was lower than that of Asp^346^. Generally, the exchange of Asp (an acidic amino acid) to His led to loss of a carboxy group and the introduction of two amidogens, while the replacement of Ala with Pro led to loss of an amidogen and the introduction of a carboxy group. Ala lacks a bulky side chain and therefore would likely not have any steric or electrostatic effects, and this change would not destroy the conformation of the main chain [7]. Differences in structures and properties among the sequences may be the reason for the differences in CoAT activity [33]. These results demonstrate that the conserved motif of CPB6-CoAT is directly linked to enzymatic activity. However, the effects of other residues on enzyme activity require further study to elucidate the function of the conserved motif in CPB6-CoAT. Clarification of the precise enzymatic mechanisms underlying enzyme binding of the butyryl-CoA or caproyl-CoA substrates might require crystallographic analyses.

In conclusion, these results confirmed the existence of a CCoAT involved in the production of caproic acid, and the enzyme is apparently different from the BCoAT responsible for the production of butyric acid. The present study improves our understanding of the metabolic reactions underlying chain elongation via the reverse β-oxidation pathway. However, determination of the detailed CCoAT structure and its function in MCFA biosynthesis require further study through protein crystallization and X-ray crystal structure analyses.

## Supporting Information

The Supporting Information is available free of charge on the Publications website.

- PCR of the CPB6-CoAT gene (encoding a CCoAT) and identification of the recombinant plasmid are shown in Supplementary Figure S1.
- PCR of the BEY8-CoAT gene (encoding a BCoAT) and identification of the recombinant plasmid are shown in Supplementary Figure S2.
- Western blot analysis of CPB6-CoAT (a CCoAT) and BEY8-CoAT (a BCoAT) is shown in Supplementary Figure S3.
- The double-reciprocal enzyme kinetics (Lineweaver–Burk) plot is shown in Supplementary Figure S4.
- The phylogenetic tree of CoATs from different strains is shown in Supplementary Figure S5.
- The sequencing peak diagram of site-directed mutant and wildtype CPB6-CoAT is shown in Supplementary Figure S6.
- The comparison of CoA-transferase activities (Mutant 1, D346H-mutant; Mutant 2, A351P-mutant) is shown in Supplementary Figure S7.
- The initial velocity of the reaction in different samples at different substrate concentrations is shown in Supplementary Figure S8.

## Supplementary Material

Supplementary Figures S1-S8

## Data Availability

All data generated or analyzed during the present study are included in this article and its supporting information.

## Abbreviations

BCoAT: butyryl-CoA:acetate CoA transferase
CCoAT: caproyl-CoA:acetate CoA transferase
CoAT: coenzyme A transferase
*k*_cat_: catalytic constant
LB: Luria broth
MCFA: medium-chain fatty acid
3D: three-dimensional
