# Supplementary material for: Butyryl/Caproyl-CoA:Acetate CoA-transferase: cloning, expression and characterization of the key enzyme involved in medium-chain fatty acid biosynthesis
Source: Biosci Rep. 2021 Aug 12;41(8):BSR20211135. doi: 10.1042/BSR20211135 (PMC8360832; doi:10.1042/BSR20211135)
Supplement: Supplementary Figures S1-S8 [file BSR-2021-1135_supp.pdf]

Supporting Information:

Butyryl/Caproyl-CoA:Acetate CoA-Transferase: Cloning, Expression and Characterization of the Key Enzyme Involved in Medium-Chain Fatty Acid Biosynthesis

Qingzhuoma Yang[1,2,3], Shengtao Guo[2,3,4], Qi Lu[1,2], Yong Tao[1,4,*], Decong Zheng[1,2], Qinmao Zhou[1,2], Jun Liu[4]

[1] Key Laboratory of Environmental and Applied Microbiology, Environmental Microbiology Key Laboratory of Sichuan Province, Chengdu Institute of Biology, Chinese Academy of Science, Chengdu 610041, China

[2] University of Chinese Academy of Sciences, Beijing 100049, China

[3] Key Laboratory of Bio-Resource and Eco-Environment of Ministry of Education, College of Life Sciences, Sichuan University, Chengdu, Sichuan, P. R. China

[4] BGI Education Center, University of Chinese Academy of Sciences, Shenzhen 518083, China

[5] Faculty of Bioengineering, Sichuan University of Science & Engineering, Xueyuan Street 180[#], Huixing Rd. 643000, Zigong, P.R. China

* To whom correspondence should be addressed. Email: taoyong@cib.ac.cn.

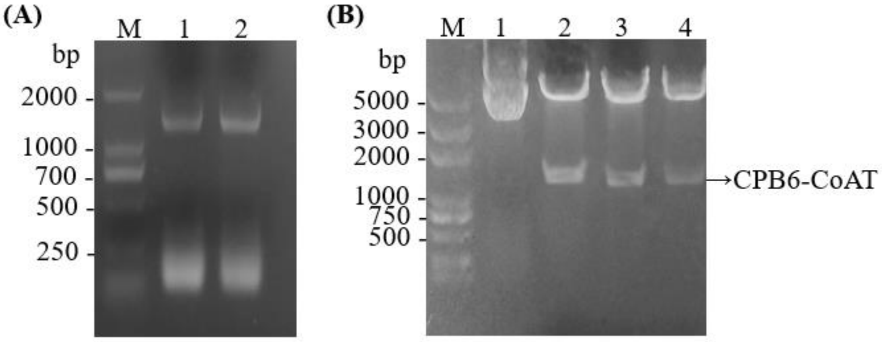

Figure S1. PCR of the CPB6-CoAT (a CCoAT) gene and identification of the recombinant plasmid

PCR amplification of CCoAT gene fragments **(A)**, M, DL2000 DNA marker; lanes 1 and 2, PCR fragments of the CCoAT from strain CPB6. Identification of the recombinant plasmid pET28a-CCoAT **(B)**. M, DL5000 DNA marker; lane 1, plasmid pET28a; lanes 2, 3 and 4, the recombinant pET28a-CCoAT plasmid double digested with *Not* I and *Sal* I.

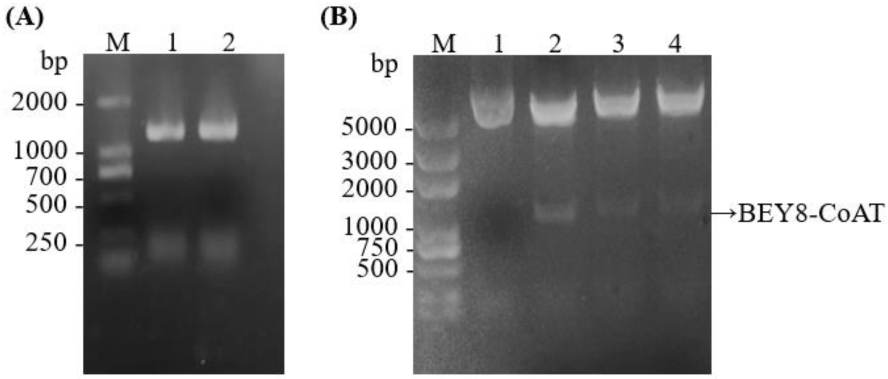

Figure S2. PCR of the BEY8-CoAT (a BCoAT) gene and identification of the recombinant plasmid

PCR amplification of BCoAT gene fragments **(A)**, M, DL2000 DNA marker; lanes 1 and 2, PCR fragments of the BCoAT from strain BEY8. Identification of the recombinant plasmid pET28a-BCoAT **(B)**. M, DL5000 DNA marker; lane 1, plasmid pET28a; lanes 2, 3 and 4, the recombinant pET28a-BCoAT plasmid double digested with *Not* I and *Sal* I.

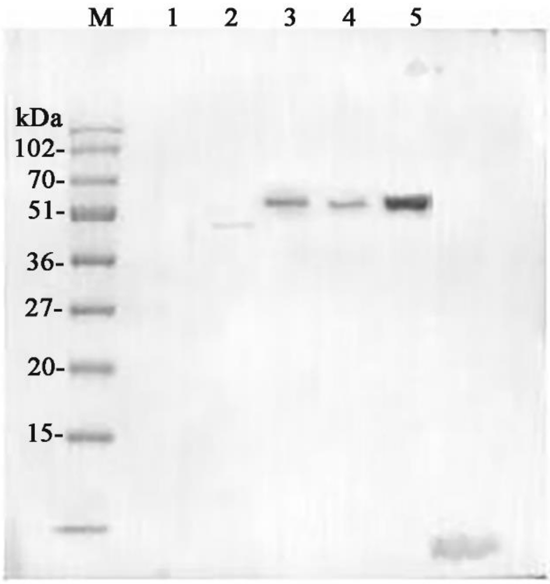

Figure S3. Western blot analysis of CPB6-CoAT (a CCoAT) and BEY8-CoAT (a BCoAT)

M, molecular mass marker. Lanes: 1, pET28a; 2, BCoAT; 3, CCoAT; 4, CCoAT-D346H mutant; 5, CCoAT-A351P mutant. Molecular mass positions are shown by markers (kDa).

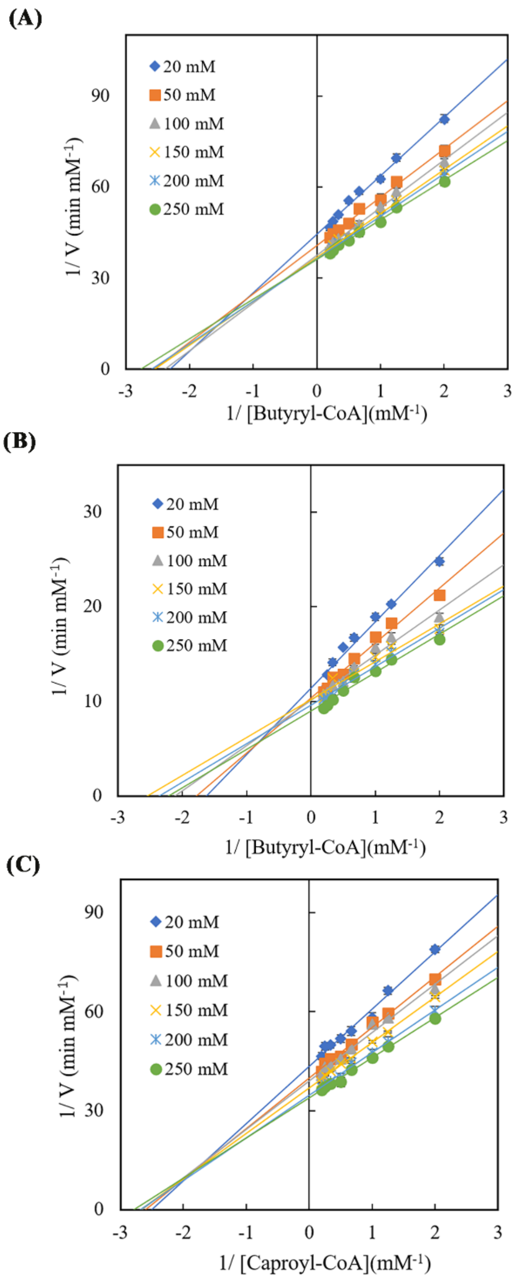

Figure S4. Double-reciprocal enzyme kinetics (Lineweaver-Burk) plot

Double-reciprocal plots of the initial velocities of acetyl-CoA and butyrate formation from butyryl-CoA and sodium acetate catalyzed by BEY8-CoAT (A) and CPB6-CoAT (B) and caproate formation from caproyl-CoA and sodium acetate catalyzed by CPB6-CoAT (C) using purified recombinant enzymes. Different butyryl-CoA and caproyl-CoA concentrations (0.5 – 5 mM) were assayed at fixed sodium acetate concentrations (20 mM, filled rhombus; 50 mM, filled squares; 100 mM, filled triangles; 150 mM, error shapes; 200 mM, star-like shapes; or 250 mM, filled circles). Data represent the mean ± standard deviation (n = 3).

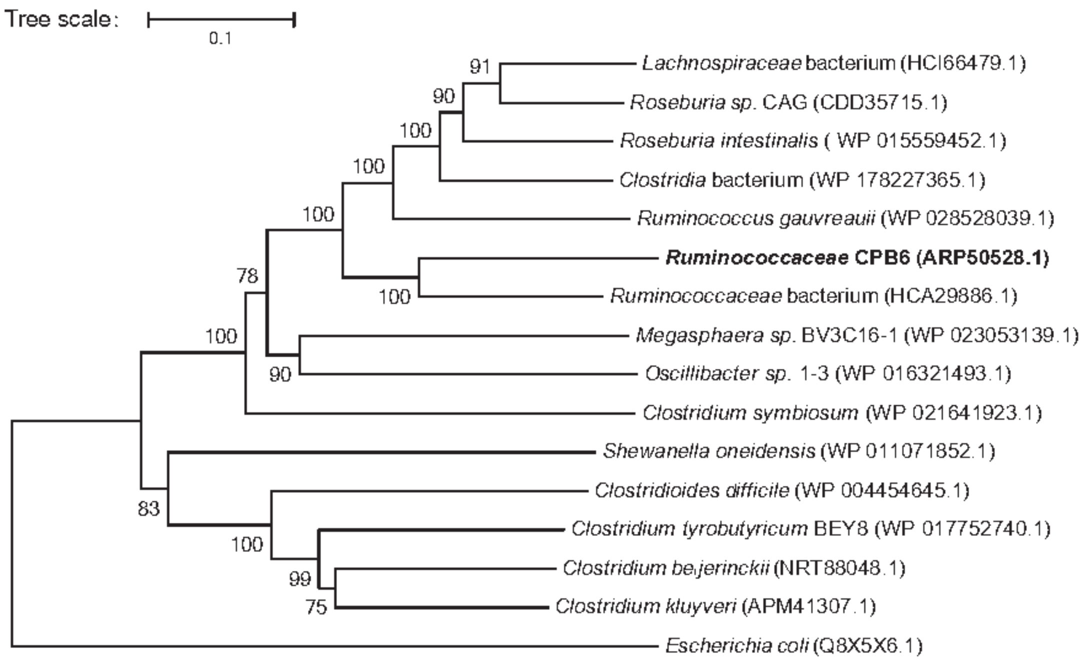

Figure S5. Phylogenetic tree of CoATs from different strains

The numbers at the nodes indicate the level of bootstrap values. The scale bar of the tree scale represents a distance of 0.1 substitutions per site.

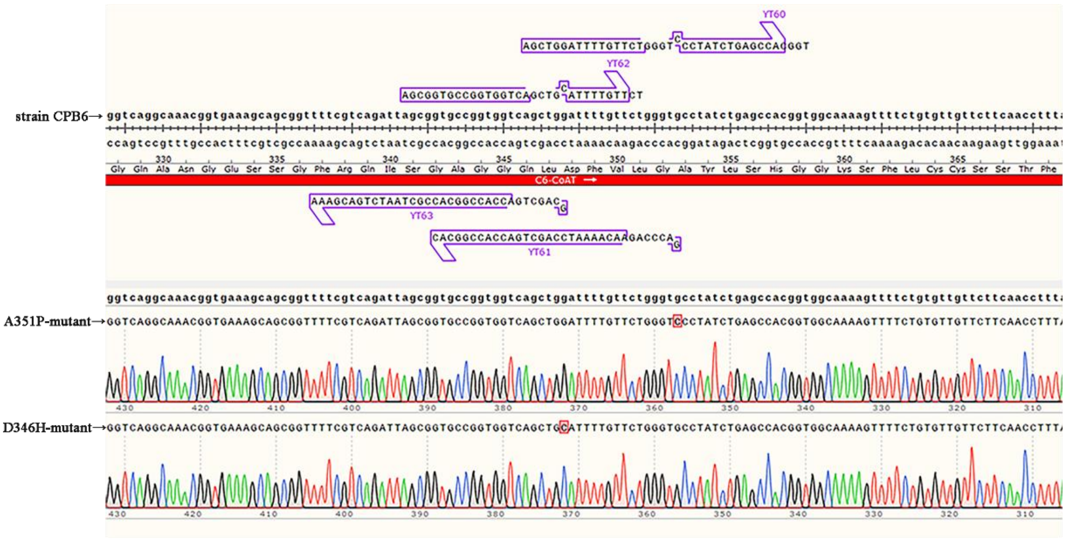

Figure S6. Sequencing peak diagram of site-directed mutant and wild-type CPB6-CoAT

Mutant nucleobases are shown in red boxes. The DNA sequences were aligned using SnapGene 2.3.2 software.

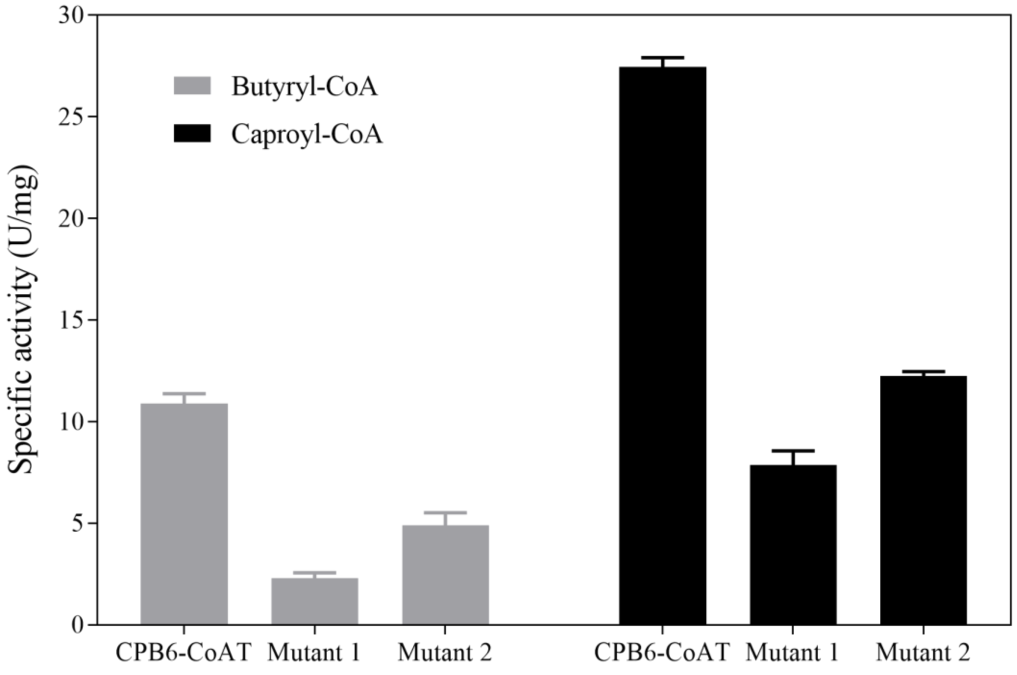

Figure S7. Comparison of CoA-transferase activities (Mutant 1, D346H mutant; Mutant 2, A351P mutant)

The specific activity with butyryl-CoA as the substrate is labeled in light gray, and the specific activity with caproyl-CoA as the substrate is marked in dark gray. The values represent the means ± SDs of three independent experiments.

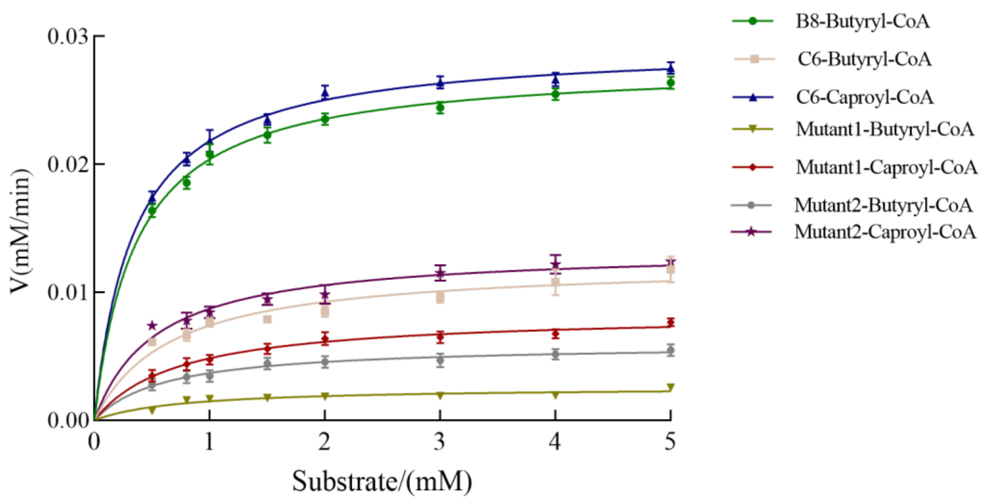

Figure S8. The initial velocity of different samples at different substrate concentrations

The V (mM/min) of the initial reaction rate was determined when the concentration of sodium acetate was fixed at different butyryl-CoA or caproyl-CoA concentrations and when the concentration of butyryl-CoA or caproyl-CoA was fixed at different sodium acetate concentrations. The values represent the means ± SDs of three independent experiments.
